# Successfully conservative management of the uterus in acute pulmonary embolism during cesarean section for placenta previa: a case report from Tu Du Hospital, Vietnam and literature review

**DOI:** 10.1186/s12245-024-00587-4

**Published:** 2024-01-29

**Authors:** Anh Dinh Bao Vuong, Thanh Hai Pham, Van Hoang Bui, Xuan Trang Nguyen, Ngoc Bich Trinh, Yen Oanh Ngoc Nguyen, Dang Khoa Tran Le, Phuc Nhon Nguyen

**Affiliations:** 1 Department of High-Risk Pregnancy, Tu Du Hospital, 284 Cong Quynh, Pham Ngu Lao Ward, District 1, Ho Chi Minh City, 71012 Vietnam; 2 Tu Du Clinical Research Unit (TD-CRU), Tu Du Hospital, Ho Chi Minh City, Vietnam; 3 Integrated Planning Room, Tu Du Hospital, Ho Chi Minh City, Vietnam

**Keywords:** Acute pulmonary embolism, Cardiopulmonary collapse, Cesarean section, Obstetrical anesthesia, Emergency, Placenta previa, Postpartum hemorrhage, Mortality

## Abstract

**Background:**

Cardiopulmonary collapse is a catastrophic event in cesarean section, which leads to adverse outcomes for both the mother and the fetus. Pulmonary embolism is one of the rare etiologies of this entity. We herein reported the successful management of acute embolism pulmonary associated with cesarean delivery on a healthy pregnant woman at our tertiary referral hospital.

**Case presentation:**

A full-term pregnant woman hospitalized for planned cesarean delivery due to placenta previa without cardiorespiratory diseases. She was scheduled uneventfully for a planned cesarean section. After placental delivery, the patient spontaneously fell into cardiopulmonary collapse and her vital signs deteriorated rapidly. The obstetricians promptly completed the cesarean section and performed all procedures to prevent the PPH and preserve the uterus. At the same time, the anesthesiologists continued to carry out advanced heart-lung resuscitation in order to control her vital signs. After surgery, the multidisciplinary team assessed the patient and found a thrombus in her pulmonary circulation. Therefore, the patient was managed with therapeutic anticoagulation. The patient recovered in good clinical condition and was discharged after 2 weeks without any complications.

**Conclusions:**

The diagnosis of acute pulmonary embolism is extremely difficult due to uncommon occurrence, sudden onset, and non-specific presentation. Awareness of this life-threatening pathology during cesarean delivery should be raised. Interdisciplinary assessment must be essentially established in this life-threatening condition. After the whole conventional management, uterine conservation may be acceptable where applicable. Further data is required to encourage this finding.

**Supplementary Information:**

The online version contains supplementary material available at 10.1186/s12245-024-00587-4.

## Introduction

Both cardiopulmonary collapse and sudden cardiorespiratory arrest are unusual during delivery. They are even more life-threatening in low-resource settings [1, 2]. Proper management of the critically ill patient in cardiopulmonary collapse requires rapid identification of its etiology. Etiologies include obstetric and non-obstetric events [3, 4]. In the obstetric field, cardiopulmonary collapse may be caused by pulmonary embolism (PE) or amniotic fluid embolism (AFE) during labor. Some risk factors include abdominal surgery, obesity, hypertension, severe preeclampsia, chronic medical diseases, and prolonged immobilization [5]. Hypercoagulability in pregnancy has been estimated to increase the risk of venous thromboembolism by about fivefold. However, it is infrequent among healthy women [6, 7]. The incidence of venous thromboembolism (VTE) was 0.4 per 1000 pregnancies, of which 83.3% were deep vein thrombosis and 16.7% were pulmonary embolism in Chinese pregnant women [8]. According to the report of Morikawa and others, the incidence of VTE after cesarean delivery (0.0074%) was significantly higher than that after vaginal delivery (0.0012%) [9, 10]. Due to its rarity, awareness of this pathology is low among reproductive-age and pregnant women, particularly, in low-middle-income countries (LMICs) [11]. APE is often rapidly fatal [12, 13]. A delayed recognition could lead to maternal death, but a misdiagnosis results in overtreatment and neglect of other pathologies [14]. According to a report of Elgendy, the rates of in-hospital mortality were almost 200-fold higher among those who had APE (29.3 vs 0.13, per 1000 pregnancy-related, *P* < 0.001) and the rates of in-hospital mortality have not improved (2.6% in 2007 vs 2.5% in 2015, *P*_trends_ = 0.74) [15]. If APE occurs before delivery, both maternal–fetal outcomes are dramatically poor [16]. Clinically, the prognosis of PE is better than AFE. PE can be treated with timely intervention but requires early recognition. PE contributes highly to peripartum maternal death [17, 18]. The suspicion of PE can be confirmed by imaging modalities, Wells score, D-dimer, and histopathological endpoints [19, 20].

PE can occur before delivery, during delivery, and in the postpartum period [16, 21]. However, the rarest is PE during cesarean section (C-section). Optimal management requires timely recognition, and treatment requires a multidisciplinary team which involves the coordinated action of the cardiologist, obstetrician, anesthesiologist, and surgeon. An implemented treatment includes thrombolysis and anticoagulation [22]. Early surgical embolectomy and bridged with extracorporeal membrane oxygenation may be required in some severe cases [23–25]. Cesarean hysterectomy is often undertaken due to the massive active bleeding after failed conservative treatment, coagulation disturbances, and a high risk of postpartum hemorrhage (PPH) that treatment of PE requires [26]. Nevertheless, hysterectomy is not optimal for a young woman who desires to preserve fertility. Meanwhile, the paucity of strong evidence in the medical literature makes it difficult to assess the safety of conservative treatment in this emergency.

Hereby, we present an uncommon case of acute embolism pulmonary during cesarean section in an otherwise healthy woman and thereby contribute to the literature on the possibility of uterine preservation where applicable.

## Case presentation

A 36-year-old woman, Gravida 3 Para 1, spontaneous abortion 2, was admitted to Tu Du Hospital due to spontaneous labor with vaginal bleeding sign. The patient’s medical history was otherwise unremarkable. Her obstetric history recorded a previous cesarean section for breech presentation and two miscarriages. At hospitalization, the patient had normal vital signs. She was at term after an uncomplicated gestation except for placenta previa Grade I. She had a known abnormal placentation site at 28 weeks of gestational age.

Therefore, an emergency C-section was scheduled. Before surgery, the patient was normotensive with a pulse of 82 beats per minute (bpm) and respiratory rate of 20 times per min. Her pre-pregnancy body mass index (BMI) was 21.6 kg/m^2^. In this pregnancy, her BMI was 25.6 kg/m^2^ (maternal weight: 54 kg, maternal height: 145 cm). Her legs were noted without gross varicose veins. Complete blood count was normal; coagulation parameters included prothrombin time (PT) of 98%, international normalized ration (INR) of 1.01, temps de quick (TQ) of 13.4 s, activated partial thromboplastin time (APTT) of 28.8 s, and plasma fibrinogen of 480 mg/dL. Electrocardiogram (ECG) showed sinus tachycardia of 116 bpm. The chest radiograph was normal. Obstetric examination revealed infrequent episodes of mild uterine contraction, a closed cervix, and intact membranes. Ultrasound showed normal fetal development in transverse lie, with an estimated weight of 3100 g with normal Doppler blood flow. The cardiotocography (CTG) proceeded as Group I by the American College of Obstetricians and Gynecologists criteria (ACOG 2009). The airway was Mallampati grade II.

At the beginning of the cesarean delivery, her blood pressure was at 130/80 mmHg, pulse rate was 100 bpm, and peripheral transcutaneous oxygen saturation (SpO2) was 100%. The patient underwent general anesthesia with tracheal intubation. After 10 min of surgery, a healthy male newborn weighing 3100 g was delivered with reported Apgar scores of 7 and 8 at 1 and 5 min, respectively, and a normal placenta was gently extracted. Transparent amniotic fluid was approximately 500 ml. However, the patient’s cardiopulmonary system collapsed immediately after placental extraction. At this time, her vital signs deteriorated with blood pressure (BP) at 90/60 mmHg, tachycardia at 125 beats/min, and SpO2 suddenly dropping to 86%. Shock index (SI) of 1.4 (0.5–0.7). Therefore, the initial differential diagnosis was amniotic embolism versus acute pulmonary embolism (Table 1). A “Red Code” was called by anesthesia while the obstetricians continued to manage the surgery. A multidisciplinary team managed the resuscitation. She responded to a total of 30 mg doses of ephedrine.

**Table 1:** Wells criteria for clinical probability assessment of pulmonary embolism

**Wells criteria/points**	**Our patient**
Clinical signs and symptoms of DVT = 3	3
An alternative diagnosis is less likely than PE = 3	3
Heart rate more than 100 = 1.5	1.5
Immobilization for 3 or more consecutive days or surgery in the previous 4 weeks = 1.5	0
Previous objectively diagnosed PE or DVT = 1.5	0
Hemoptysis = 1	1
Malignancy (on treatment, treatment in last 6 months, or palliative) = 1	0
**Total**	8.5
**Traditional clinical probability assessment (Wells criteria)**	
0–1 = low risk 2–6 = moderate risk > 6 = high risk	High risk
**Simplified clinical probability assessment (Modified Wells criteria)**	
PE likely = > 4.0 PE unlikely = ≤ 4.0	PE likely

*DVT* deep vein thrombosis, *PE* pulmonary embolism

During surgery, the obstetrician focused on hemostasis carefully. Bilateral artery ligation, placental bed sutures, and B-Lynch compression sutures were all performed due to the high risk of PPH. Uterotonic drugs including oxytocin, methyl ergonovine, carboprost, and tranexamic acid were administered. The uterus was contracted, so the team decided to preserve the uterus. The uterus and abdomen were quickly and carefully closed to minimize the surgical time. An abdominal drain was placed. Total estimated blood loss was measured at 500 ml and surgical time duration was 100 min. Urine output was measured at 100 ml during surgery.

**Fig. 1:**
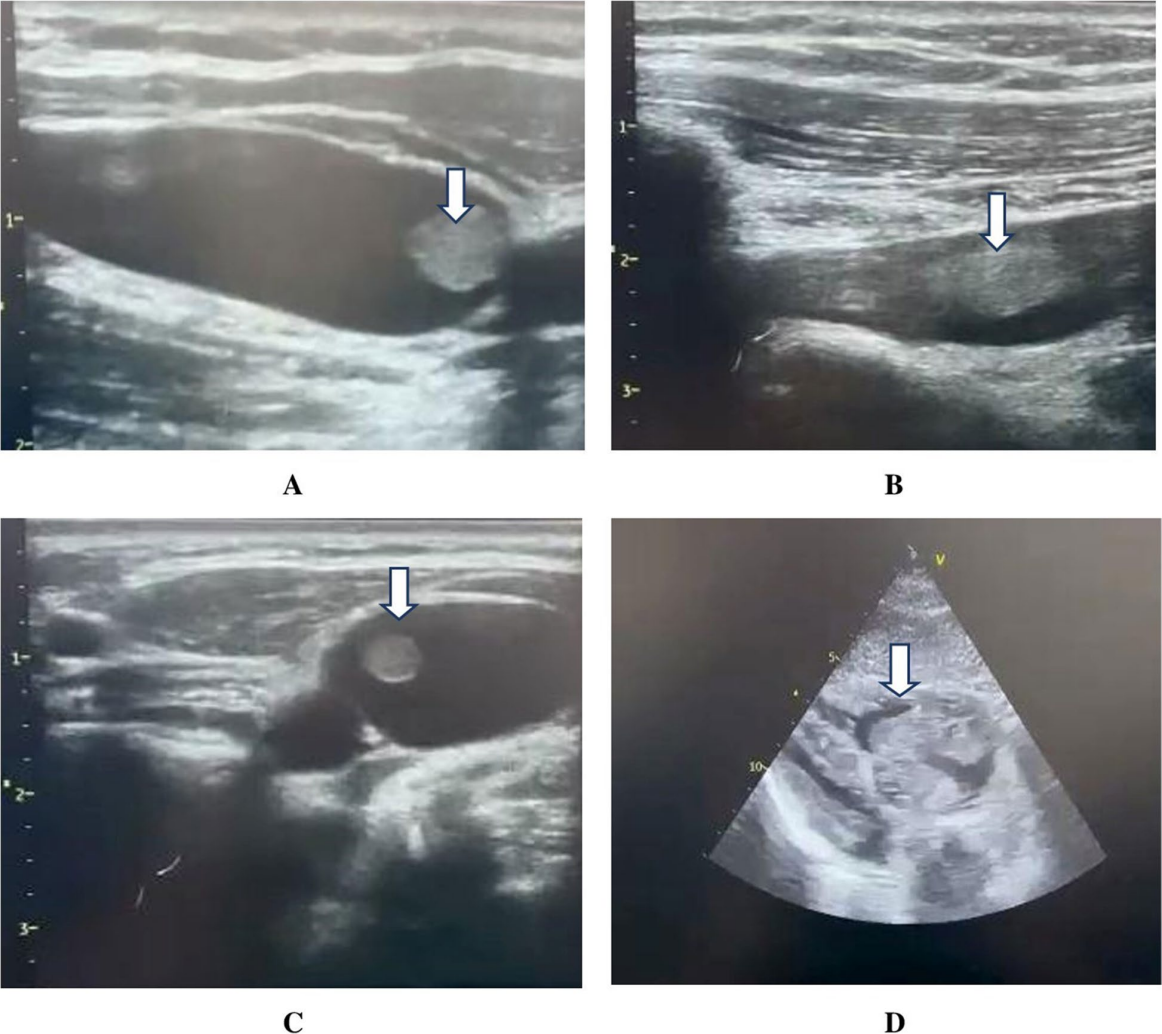
Ultrasonic findings showed a sizable thrombus (white arrow) in the pulmonary vascular (**A**), in the subclavian vein (**B**), in the right jugular vein (**C**), and in the right ventricular (**D**), respectively

Meanwhile, urgent transthoracic echocardiography showed a sizable thrombus in the pulmonary vasculature, in the subclavian vein, in the right jugular vein, and in the right ventricular cavity (Fig. 1 and Supplementary videos 1, 2, 3, and 4, respectively). Thus, a diagnosis of pulmonary embolism was established. Ultrasonic assessment demonstrated no abnormalities in the lower limb veins.

After surgery, the patient was transferred immediately to the intensive care unit (ICU) where she was cared for by a multidisciplinary team consisting of obstetrician, anesthetist, cardiologist, hematologist, and sonographer. Ventilation was maintained by a positive pressure-supported respirator. She was sedated, paralyzed, and maintained with a narcotic, an anticonvulsant drug, and a muscle relaxant drug (Fentanyl 100 mg 02 ampules combined with Midazolam 5 mg 05 ampules intravenous infusion at 6 ml/h, with Rocuronium 10 mg/ml × 5 ampules in 45 ml sodium chloride 0.9% for 50 ml infused via an intravenous electric pump at 10 ml/h). Additional analgesia included subcutaneous morphine sulfate 10 mg and diclofenac 100-mg rectal suppository.

D-dimer levels were markedly increased to 46,700 ng/ml. The coagulation profile showed a weak to moderate blood clot contraction with PT = 62%, INR = 1.29, TQ = 13.8 s, TCK = 34 s, and fibrinogen = 136 mg/dl. Hemoglobin level fell to 7.6 g/dl. The patient was transfused with 2 units of AB-positive packed red blood cells (350 ml/unit) and was given 4 packs of fresh frozen plasma (200 ml/unit), as well as all 10 packs of cryoprecipitate (50 ml/unit). The patient received a therapeutic dose of one ampule of low-molecular-weight heparin (25,000 IU/5 ml/ampule). The abdomen drained 200 ml of abdominal cavity fluid without active bleeding. An intravenous broad-spectrum antibiotic (tarzocin 4.5 g/ampule every 6 h) was given to prevent the secondary infection.

After counseling with an interdisciplinary team, the patient was transferred to Cho Ray Hospital, a tertiary general hospital for further management after 16 h of obstetric and hemodynamic stabilization. At the tertiary center, chest X-ray was normal, and cardiac ultrasound revealed mild right ventricular dilation and normal left systolic function. Unfortunately, the computed tomographic pulmonary angiography (CTPA) confirmed nearly total occlusion of segmental arterial branches from the upper lobes of both lungs, the left and the right lower lobar branches. The descending branch of the left pulmonary artery was partially occluded; the lateral basal and the posterior basal arterial branches of the left lower lobe were completely occluded. Both lungs had small pleural effusions and passive atelectasis of inferior areas. Abdominal ultrasound showed free air, infiltration, and a small amount of fluid in the lower right abdomen. The hepatic and renal function tests as well as the electrolyte profile were within normal limits. However, high sensitivity Troponin 1 and NT-proBNP were elevated at 637.5 pg/mL and 96.75 pmol/L, respectively. The complete blood count test showed moderate anemia with Hb of 79 g/L (7.9 g/dL), an increased white blood cell count (WBC) of 17.69 G/L, and a low platelet count of 119 G/L.

At the tertiary hospital, the patient received 2 additional units of blood and was intensively treated with anti-coagulant therapy. Specifically, heparin 10,000 IU was first administered with 50 ml sodium chloride 0.9% solution, giving a bolus of 15 ml (3000 IU) and maintenance of 4–6 ml per hour (18 IU/kg) monitored by activated clotting time (ACT) levels (147–172 s). On the second day, the patient was extubated; oxygenation was supported with a nasal cannula. Heparin was stopped. Lovenox 60 mg/0.6 ml was subcutaneously injected twice a day at 12-h intervals. Additionally, she received pantoprazole for gastroesophageal reflux disease (GERD) during her admission to the intensive care unit. She was discharged after 2 weeks in good health, with normal consciousness and without neurological injuries. The patient and her family felt grateful to the team for saving her life.

## Discussion

Cardiopulmonary complications of C-sections are a leading cause of maternal morbidity and mortality, particularly in low-middle-income countries or in low-resource settings [2]. Almost all cases of thromboembolism (TE) are diagnosed during the postpartum period. An APE that occurs immediately during C-section seems rarely to be reported. This pathology is mainly documented through a case by case in the literature [7, 27, 28]. According to the retrospective study of Lai et al., out of 1377 pregnant women who underwent C-section, only 7 cases developed venous TE and 86% of cases were within 7 days of postpartum. This study revealed that hypertension and the presence of varicose veins were associated with TE following C-section [29]. In our case, several risk factors contributed to this woman’s cardiorespiratory collapse. First, an advanced maternal age > 35 years old, previous cesarean delivery, and C-section due to placenta previa in this pregnancy (Table 2). After the coronavirus disease 2019 (COVID-19) pandemic, the incidence of VTE seems likely to increase [30–32]. Particularly, the PE cases associated with the placental implantation site have been recorded recently at our center. Placenta previa resulting in a massive PPH may be a high risk for VTE [33, 34]. However, none of the similar reports has been documented in the literature. Therefore, the underlying mechanism of this occurrence due to hidden blood clots from the deep veins before surgery or from placenta previa after placental delivery remains controversial.

**Table 2:** Summary of peripartum pulmonary embolism over the past last 10 years in the literature

Authors/year	Maternal age (yo) Gravida, parity	Gestational age	Medical history, risk factors	Mode of birth delivery	Indication for CS	Mode of anesthesia	Presentation/Onset	Management	Hypothesis	Identified diagnosis	Outcomes
Ho et al. (2014) [25]	37 yo G2P1	37w 2d	None	Emergent CS	Nonreassuring fetal heart rate	-	-Bilateral limb weakness, consciousness disturbance -Unstable hemodynamic status, hypotension (46/20 mmHg), tachycardia (121 beats/minute), low oxygen saturation (85%), tachypnea (32 breaths/minute), hypoxemia, and respiratory alkalosis	-CPR - Venoarterial ECMO - Emergent catheter-directed thrombectomy - Continuous low molecular weight heparin (LMWH) infusion, stopped later due to hemoperitoneum hemorrhage	-	Massive PE	- Alive - Full recovery - Transferred to a general ward for observation
Van Liempt et al. (2015) [35]	40 yo	35w 2d	none	CS	Vaginal bleeding/placenta previa	Spine anesthesia	Asystole occurred during uterotomy	CPR and fetal delivery	-Bezold Jarisch reflex -Amniotic fluid embolism -Venous air embolism	None	-Recovery in 48 h -Alive
Wang et al. (2015) [36]	24 yo	40w 4d	none	-VB -PAS Conservative management with uterine arterial embolism, hysteroscopic resection, and mifepristone	-	-	-Dyspnea and loss of consciousness -Acute respiratory distress syndrome	CPR	-	Acute trophoblastic PE and allergic shock when infusing povidone-iodine	Death
Yufune et al. (2015) [37]	38 yo	38w 1d	Frequent transient ischemic attacks during hyperventilation associated with Moya Moya disease	CS	A previous cesarean section and Moya Moya disease	General anesthesia	-Massive vaginal bleeding without clotting - Disseminated intravascular coagulation -Hypovolemic shock -Cardiac arrest	-Blood volume replacement -Coagulation therapy (fresh frozen plasma, platelets, fibrinogen, antithrombin concentrate) -Emergency relaparotomy -CPR	-	Amniotic fluid embolism	alive
Pandy et al. (2015) [27]	35 yo	-	Obese	CS	-	-	A syncopal attack following cesarean delivery	-	-	Pulmonary embolism	-
Colombier et al. (2015) [23]	36 yo	36w	None	Emergent cesarean delivery	Pathologic cardiotocography after spontaneous membrane rupture	Epidural anesthesia	-Hemodynamically unstable, presenting severe bradycardia and hypotension, followed by cardiac arrest and active intra-uterine bleeding after 30 min from CS -Echocardiography revealed a severe right heart dysfunction and massive dilatation - CT scan of the lungs confirmed the diagnosis of PE and showed an occlusion of the segmental and sub-segmental pulmonary arteries	-CPR -Emergency surgical pulmonary embolectomy -Followed by a hysterectomy	-	massive bilateral PE	-Alive -Follow-up at 3 months showed a persistent right ventricular dilatation and moderate dysfunction -Patient complained a persistent slight dyspnea at physical effort (NYHA II)
Ahn et al. (2016) [38]	35 yo	Term	None	CS	-	-	-Dyspnea -Hypotension in 24 h after CS	Embolectomy	-	Massive bilateral pulmonary thromboembolism	Alive
Umazume et al. (2017) [39]	28 yo	37w 3d	BMI 23.6 kg/m^2^	CS	Placenta previa	Combined spinal and epidural analgesia	Hypoxemic	CPR	Amniotic fluid embolism	Transient bronchospasm and pulmonary hypertension	Alive
Oda et al. (2018) [40]	25 yo	38w 4d	none	CS	Repeat CS	Spinal anesthesia	Dyspnea, hypotension, and loss of consciousness with decreased peripheral oxygen saturation after removal of the placenta	- Tracheal intubation and mechanical ventilation with oxygen -Heparin	-	Pulmonary embolism caused by ovarian vein thrombosis extending up to the inferior vena cava	-Recovery in 1 day -Alive
Tong et al. (2019) [41]	27 yo	40w	Residual placenta	VB	-	-	Fever and dyspnea after delivery	-Antibiotics -Low molecular weight heparin -Warfarin -Mifepristone, then hysteroscopy	-	Pulmonary embolism	Alive
Finianos et al. (2021) [42]	37 yo Multiparous	31w	Ovarian vein thrombophlebitis Uterine fibroid	CS	Repeat CS following preterm rupture of membranes	-	Severe abdominal pain, fever, and chills	-Therapeutic anticoagulation with low molecular weight heparin - Antibiotic	-	Subsegmental pulmonary embolism	Alive
Tiwary et al. (2022) [43]	37 yo	-	BMI = 28 kg/m^2^ American Society of Anesthesiologist physical status II	Emergency LSCS	-	-	Desaturation Tachypnea	Therapeutic anticoagulation using low-molecular-weight heparin (enoxaparin)	-	Bilateral pulmonary embolism	alive
Wu et al. (2022) [44]	32 yo	39w 6d	IVF subclinical hypothyroidism	CS	Requirement	-	-Shortness of breath after activity after 14 days of delivery -D-dimer was 7440 ng/mL	-Anticoagulation with low molecular weight heparin (LWMH)	-	Pulmonary embolism	-Recovery in 1 week -Alive
Wu et al. (2022) [44]	-	37w 4d	GDM; breast fibroma; recurrent shortness of breath	CS	Requirements and chest tightness	-	Paroxysmal chest tightness, shortness of breath, discomfort, slight cough after 18 days of delivery - D-dimer was 1500 ng/mL	-Anticoagulation therapy immediately by subcutaneous injection of Enoxaparin 4100 IU twice daily	-	Pulmonary embolism	Alive
Zhang et al. (2022) [45]	25 yo Nulliparous	40w 4d	None	Emergency CS	Retention of fetal head descending and persistent occipito-posterior position	Combined spinal-epidural anesthesia	Cough hypotension, tachycardia, hypoxemia, dyspnea, cyanotic after the end of CS 2 min	Resuscitation	Amniotic-fluid embolism	-	Alive
Karakosta et al. (2023) [26]	39 yo G5P2	37w 5d	BMI 26.5 kg/m^2^ ASA II	CS	Repeat cesarean section	General anesthesia	Sudden drop in end-tidal CO2 after placenta delivery combined with significant hemodynamic instability	-Thrombolysis by recombinant tissue plasminogen activator under continuous -US monitoring, Bakri balloon placement, and rescue hysterectomy	-	Acute pulmonary embolism	Alive with the removal of the uterus
Zawislask et al. (2023) [46]	40 yo G8P9	39w	none	CS	Pulmonary embolism	General anesthesia	Dyspnea, shortness of breath, and chest pain Hypotension Tachycardia Hypoxemia, tachypnea, high D-dimer levels of 17,189 ng/ml before CS	-Unfractionated heparin monitored with activated partial thromboplastin time -CPR -Emergency pulmonary embolectomy in extracorporeal circulation	-	Massive central pulmonary embolism	-Recovery in 3 days - Alive
Song et al. (2023) [28]	31 yo G1P1	39w 4d	A dilated left ventricle with a patent foramen ovale	Planned CS	Macrosomia and separation of the symphysis	Spinal anesthesia at L3 to L4	-Dyspnea and dull pain in the left back after surgery -Significantly elevated D-dimer (4.359 mg/L) - a blood clot in the left common iliac vein	-Low-molecular-weight heparin -Catheter-directed thrombus fragmentation and thrombolysis -combined anticoagulant therapy	-	Postpartum pulmonary embolism from iliac vein thrombosis	-Alive -Recovery after 6 months of follow-up
Park et al. (2023) [24]	36 yo G3P2, once CS	35w 4d	Obesity (BMI = 34.6 kg/m^2^)	Emergent CS	Fetal tarchycardiac	General endotracheal anesthesia	-Drowsy, SpO2: 77% - Cardiac arrest - CT pulmonary angiography after cardiopulmonary securement was performed to confirm PE	- CPR - VA ECMO - surgical thrombectomy	-	Massive PE	-Alive -Discharge on day 50 - Follow-up was stopped 20 months after thrombectomy
Krawczyk et al. (2023) [7]	34 yo G3P2	24w4d Dichorionic twin pregnancy	WHO class III obesity (BMI = 44 kg/m^2^)	CS	Subchorionic hematoma and suspicion of placental abruption	General anesthesia	-An episode of sinus tachycardia (160 bpm) with a blood pressure drop to 90/50 mmHg -Cardiac arrest was confirmed 10 min after the delivery - Uterine atony and severe hemorrhage	- CPR - Heparin i.v -Postpartum hysterectomy -Blood transfusion	-Massive pulmonary embolism - Amniotic fluid embolus	CT pulmonary angiography was done without filling defect suggesting pulmonary embolism	-Alive -Both mother and twin newborn were discharged on day 3
	24 yo G2P2 once CS	28w	WHO class II obesity (BMI 36.7 kg/m^2^), immobilization, thrombophilia	Emergent CS	Premature abruption of the placenta after fetal surgery for placing vesicoamniotic intrauterine shunt	Spinal anesthesia	-Dyspnea, chest pain, and presented cyanosis - Sinus tachycardia 120/min - Cardiac arrest	- Heparin i.v - CPR - Actilyse - Oral warfarin -Blood transfusion	Suspected PE	CT pulmonary angiography was done without filling defect suggesting pulmonary embolism	-Alive -Both mother and baby were sent home on day 23
Urriago-Osorio et al. (2023) [18]	24 yo G3P2	26w	-	-	-	-	- Unconscious, diaphoretic, and cold, with subsequent partial recovery of consciousness, and after collapsing three times - Stuporous, diaphoretic, and cold, with a blood pressure of 60/28 mmHg and a heart rate of 155 bpm	- Vasopressor therapy -LMWH (enoxaparin 60 mg subcutaneously every 12 h) -Thrombolytic therapy with alteplase 100 mg intravenously	Point of care ultrasound (POCUS) revealed a suspected PE	Massive PTE	recovery and alive
The present case	36 yo	-37w2d -G3P1 once CS	-Advanced maternal age -Placenta previa	Emergent CS	Labor, vaginal bleeding in pregnancy with placenta previa	General anesthesia	Sudden cardiopulmonary collapse immediate after placental delivery	-CPR -blood transfusion - Anti-coagulant therapy -Multidisciplinary assessment -Interhospital management	-AFE -APE	US and CT angiopathy showed APE	-Alive -Recovery after 2w without severe sequela

*APE* acute pulmonary embolism, *AFE* amniotic fluid embolism, *ASA* American Society of Anesthesiologists, *BMI* body mass index, *CPR* cardiopulmonary resuscitation, *CT* computed tomography, *CS* cesarean section, *d* days, *NYHA* New York Heart Association, *LMWH* low molecular weight heparin, *PAS* placenta accreta spectrum, *P* parity, *G* gravida, *VB* vaginal birth, *SpO2* saturation of peripheral oxygen, *VA ECMO* veno-arterial extracorporeal membrane oxygenation, *VB* vaginal birth, *US* ultrasound, *yo* years old, *w* weeks, *WHO* World Health Organization

A timely diagnosis of APE remains totally difficult since it is overlapped with other complications and is camouflaged by the physiological changes in pregnancy [16]. In the case of a parturient with signs of sudden cardiopulmonary arrest during C-section, the initial differential diagnosis includes anaphylaxis, pulmonary embolism (PE), and acute coagulopathy and even cerebral hemorrhage due to amniotic fluid embolism, or cerebral hemorrhage due to severe preeclampsia. However, this patient did not suffer hypertension during pregnancy. The patient showed no immediate signs of allergic reaction to the general anesthesia and the collapse did not occur immediately.

Clinically, the catastrophic manifestation of PE and AFE is similar. In the current case, since the patient was under general anesthesia, the initial clinical symptoms such as dyspnea, shortness of breath, and chest pain were absent. Diagnosis criteria in AFE have been studied [47]. According to the Society of Maternal Fetal Medicine (SMFM) and the Amniotic Fluid Embolism Foundation, the four proposed diagnostic criteria for AFE include (1) sudden cardiac arrest or both respiratory and hemodynamic collapse, (2) disseminated intravascular coagulopathy (DIC), (3) absence of fever, and (4) clinical onset during labor or within 30 min of delivery [12, 48]. Well’s diagnostic criteria for PE were met in our patient [20]. An increased D-dimer is a valuable predictive marker for PE (Table 1). The D-dimer cut-off value of 800 ng/mL ensures high sensitivity and increases specificity compared to the conventional threshold of 500 ng/mL. Considering this higher threshold can reduce the number of unnecessary CT scans and subsequently unnecessary radiation exposure in women after Cesarean delivery [49, 50]. Nevertheless, the value of D-dimer is still limited since the concentration depends on the gestational age [14]. Recently, the value of the Fibrin monomer test has also been a concern since its concentration is nearly unchanged during pregnancy [51]. However, our patient was assessed with a low risk of VTE during the antenatal period following The Royal College of Obstetricians and Gynaecologists (RCOG Green-top Guideline, no. 37a. Reducing the risk of thrombosis and embolism during pregnancy and the puerperium. London: RCOG, 2015); thus, the screening modalities were not indicated [34]. According to local guidelines, no routine thromboprophylaxis was given during the antenatal care. Moreover, the patient was in emergent condition with hemorrhage originating from placenta previa. Thus, the surgery was a priority.

But more than 90% of pregnant women with suspected PE do not have PE [14]. The gold standard for diagnosis of PE is found at autopsy. Fortunately, the patient lived, so the only evidence for PE was the ultrasonic findings of the blood clot in the heart and pulmonary artery. Ultrasound can be performed at the bedside more rapidly than a CT scan and so is a more useful diagnostic tool in a critical situation [19]. CT angiopathy can be indicated to confirm the diagnosis of APE when the patient is in stable condition [7, 24, 52].

Ultrasound can also help differentiate between PE and AFE. Histopathologically, AFE material can include amniotic fluid, as well as fetal cells, hair, or other debris entering the mother’s bloodstream through the placental bed. It is usually rapidly progressive and has a very high mortality [53]. These materials are sonographically different in appearance from a clot. In our patient, no strange structure was on ultrasound. Thus, cardiopulmonary collapse due to PE was the most likely diagnosis in this pregnant woman.

The neonatal outcome should be mentioned. In this case, PE occurred after fetal extraction. The infant was unaffected. However, when PE occurs prior to cord clamping, the wellbeing of both mother and child is at risk. Emergency C-section may be necessary to save the newborn life. Additionally prompt delivery improves maternal outcome by reducing maternal vena cava compression due to the gravida uterus, thus increasing maternal cardiac output, and may improve the hypercoagulability of pregnancy [21, 46, 53].

The modern management of PE includes anti-coagulation, thrombolysis, and surgical pulmonary embolectomy. The choice of management should be based on the patient’s status and the size of the thrombus clot [26, 38]. However, no practical guidelines have been established for this difficult entity [46]. Thrombolytic therapy could be used if the PE occurs before labor [18]. Remarkably, thrombolysis and administration of tissue plasminogen activator may be contraindicated at the onset of massive hemorrhage during surgery [23, 24]. Postpartum thrombolytic therapy with recombinant tissue plasminogen activator (rt-PA) needs to be strictly monitored because the treatment may lead to massive bleeding [25, 54]. In the present case, anticoagulant treatment using intravenous heparin and low molecular weight heparin subcutaneous injection were indicated during obstetric stabilization without excessive vaginal bleeding.

In general, it is a risk to conserve the uterus in a pregnant woman with PE because of the high risk of PPH and the associated coagulopathies. Previously, almost all reports have recommended emergent cesarean hysterectomy in the management of pulmonary embolism during cesarean delivery. Intra-uterine tamponade using a Bakri® postpartum balloon may be performed for temporary hemostasis [23]. In this case, the patient was administered 1 g of tranexamic acid as a routine drug in the PPH during C-section. The team did not assess the pulmonary embolism at the initial moment. However, the risks and benefits of this drug administration are still controversial due to the risk of thrombosis [55–57]. Karakosta et al. have recently reported a case of uterine conservation after acute life-threatening PE during C-Section which required postpartum rescue hysterectomy due to massive bleeding [26]. Initial uterine conservation carries the risk of subsequent emergency hysterectomy. Immediate resuscitation and coagulopathy monitoring is essential. Life-saving treatment must always be a priority over future fertility. However, when there is a multidisciplinary team immediately available and when immediate surgery could be undertaken if necessary, uterine conservation with preservation of future fertility can be considered. In addition, aside from uterotonic drugs, the time duration between a cesarean hysterectomy and all hemostatic procedures in uterine conservative surgery is also a main concern since it relates directly to maternal outcomes. In our case, the total surgical time duration from the onset of APE to abdominal closure was 110 min. Due to a lack of evidence, the team could not compare this surgical time to any documents in the literature. Further studies are required to clarify this entity. Nevertheless, regarding the surgical methods, the hemostatic procedures relating to this surgery were mentioned in a recent article. In this article, the total time duration of the uterine conservative surgery was shorter than that of a cesarean hysterectomy [58].

Particularly, postpartum care needs to be strictly monitored in APE cases due to the high risk of PPH and infectious post-surgery. Song et al. have reported a case of obstructive uropathy caused by a 20-cm-sized hematoma anterior to the bladder in Retzius space following postpartum pulmonary embolism [59]. Recently, elevated plasma lipocalin-2 levels have been a promising biomarker in predicting long-term major adverse events among normotensive patients with APE for risk stratification in the intermediate-risk group [60]. Most importantly, multidisciplinary management with interhospital transfer and advanced armamentarium is necessarily required to control the coagulopathy profile, prevent multi-organ dysfunction, and reduce significant mortality [23, 61]. Maintenance therapy using LMWH or warfarin should be continued for at least 6 weeks postnatal and for a minimum of 3 months in total as these have proven safe in breastfeeding women. The subsequent pregnancy needs to be treated with VTE prophylaxis [62].

## Conclusions

The diagnosis of APE remains a challenge due to its rarity, sudden onset, and non-specific symptoms. A high index of suspicion for acute pulmonary embolism during cesarean delivery is potentially necessary, even in any parturient woman. Immediate interdisciplinary resuscitation can minimize maternal death. Although cesarian hysterectomy has been the standard treatment, when the right resources are present, uterine conservation may be considered. Further data is required to determine under which conditions cesarean hysterectomy or uterine conservative management is better.

### Supplementary Information

**Additional file 1: Supplementary video 1.** Sizable thrombus in the pulmonary vascular.

**Additional file 2: Supplementary video 2.** Sizable thrombus in the subclavian vein.

**Additional file 3: Supplementary video 3.** Sizable thrombus in the right jugular vein.

**Additional file 4: Supplementary video 4.** Sizable thrombus in the right ventricular.

## Data Availability

The datasets used and/or analyzed during the current study are available from the corresponding author upon reasonable request.

## Abbreviations

AFE: Amniotic fluid embolism
APE: Acute pulmonary embolism
CS or C-section: Cesarean section
PE: Pulmonary embolism
PPH: Postpartum hemorrhage
GA: Gestational age
US: Ultrasound
VTE: Venous thromboembolism
